# Supplementary material for: Incidence and Associations of Acute Kidney Injury after General Thoracic Surgery: A System Review and Meta-Analysis
Source: J Clin Med. 2022 Dec 21;12(1):37. doi: 10.3390/jcm12010037 (PMC9821434; doi:10.3390/jcm12010037)
Supplement: Supplementary file 1 [file jcm-12-00037-s001.zip › Supplementary Data S1.pdf]

**Supplementary Data S1.** Search terms for systematic review

Cochrane

#1 (Pulmonary surgery):ti,ab,kw OR (Lung surgery):ti,ab,kw OR (thoracic surgery):ti,ab,kw OR (Esophageal surgery):ti,ab,kw

#2 (Acute Kidney Injury):ti,ab,kw OR (Acute Renal Injury):ti,ab,kw OR (AKI):ti,ab,kw OR (Acute Kidney Failure):ti,ab,kw OR (Acute Renal Failure):ti,ab,kw

#1 and #2

EMBASE

#1 'acute renal failure':ti,ab,kw OR 'acute kidney failure':ti,ab,kw OR aki:ti,ab,kw OR 'acute renal injury':ti,ab,kw OR 'acute kidney injury':ti,ab,kw

#2 'pulmonary surgery':ti,ab,kw OR 'lung surgery':ti,ab,kw OR 'thoracic surgery':ti,ab,kw OR 'esophageal surgery':ti,ab,kw

#1 and #2

PUBMED

("pulmonary surgery"[Text Word] OR "lung surgery"[Text Word] OR "thoracic surgery"[Text Word] OR "esophageal surgery"[Text Word]) AND ("AKI"[Text Word] OR "acute kidney injury"[Text Word] OR "acute renal injury"[Text Word] OR "acute kidney failure"[Text Word] OR "acute renal failure"[Text Word])
