# Supplementary material for: Incidence and Associations of Acute Kidney Injury after General Thoracic Surgery: A System Review and Meta-Analysis
Source: J Clin Med. 2022 Dec 21;12(1):37. doi: 10.3390/jcm12010037 (PMC9821434; doi:10.3390/jcm12010037)
Supplement: Supplementary file 1 [file jcm-12-00037-s001.zip › Table S1.pdf]

Table S1. Newcastle-Ottawa Scale for assessing the quality of studies in meta-analysis

Study Author	Selection	Comparability	Outcome	Overall score	Arbitrary Rating
**Licker et al**	4	0	3	7	Good
**Ishikawa et al**	4	0	3	7	Good
**Lee et al**	4	0	3	7	Good
**Ren et al**	4	0	3	7	Good
**Assaad et al**	4	0	3	7	Good
**Grams et al**	4	0	3	7	Good
**Ahn et al**	4	0	3	7	Good
**Moon et al**	4	0	3	7	Good
**Konda et al**	4	0	3	7	Good
**Wang et al**	4	2	3	9	Good
**Cardinale et al**	4	0	3	7	Good
**Naruka et al**	4	0	3	7	Good
**Matesanz et al**	4	0	3	7	Good
**Oh et al**	4	2	3	9	Good
**Garutti et al**	4	0	3	7	Good
**Murphy et al**	4	0	3	7	Good
**Meng et al**	4	0	3	7	Good
**Kim et al**	4	0	3	7	Good
**Zhao et al**	4	0	3	7	Good
**Wu et al**	4	0	3	7	Good
