# Supplementary material for: Incidence and Associations of Acute Kidney Injury after General Thoracic Surgery: A System Review and Meta-Analysis
Source: J Clin Med. 2022 Dec 21;12(1):37. doi: 10.3390/jcm12010037 (PMC9821434; doi:10.3390/jcm12010037)
Supplement: Supplementary file 1 [file jcm-12-00037-s001.zip › Table S2.pdf]

Table S2. Reported stage of postoperative AKI

Study (Year)	Definition of AKI	Number of patients with AKI	Stage 1 or RIFLE –R	Stage 2 or RIFLE – I	Stage 3 or RIFLE – F
**Licker et al (2011)**	RIFLE	91	56	33	2
**Ishikawa et al (2012)**	AKIN	67	59	8	0
**Lee et al (2014)**	AKIN	210	180	16	14
**Ren et al (2015)**	KDIGO	23	13	7	3
**Assaad et al (2015)**	AKIN	3	3	0	0
**Grams et al (2016)**	KDIGO	1413	1083	221	109
**Ahn et al (2016)**	AKIN	74	39	2	33
**Cardinale et al (2018)**	AKIN	222	200	15	7
**Naruka et al (2019)**	KDIGO	86	55	25	6
**Murphy et al (2020)**	AKIN	208	173	28	7
**Meng et al (2020)**	KDIGO	31	27	2	2
**Kim et al (2020)**	AKIN	63	54	6	3
**Zhao et al (2021)**	KDIGO	205	172	21	12

Abbreviations: AKI, Acute kidney injury; AKIN, acute kidney injury network; KDIGO, kidney disease improving global outcomes; RIFLE, risk, injury, failure, loss of kidney function, and end-stage kidney disease.
