# Supplementary material for: Incidence and Associations of Acute Kidney Injury after General Thoracic Surgery: A System Review and Meta-Analysis
Source: J Clin Med. 2022 Dec 21;12(1):37. doi: 10.3390/jcm12010037 (PMC9821434; doi:10.3390/jcm12010037)
Supplement: Supplementary file 1 [file jcm-12-00037-s001.zip › Table S3.pdf]

Table S3. Rate of postoperative mortality of patients with/without postoperative AKI

Study	Number patients with/without AKI	Morality definition	Total deaths	Deaths with AKI	Deaths without AKI	RR of AKI	P
Licker et al	91/1254	Hospital	31(2.3%)	18(19.8%)	13(1.0%)	19.08	<0.001
Ishikawa et al	67/1062	Hospital	10(0.8%)	2(3.0%)	8(0.8%)	3.96	0.12
Lee et al	210/385	30-day	18(3.0%)	10(4.8%)	8(2.1%)	2.29	0.115
Ren et al	23/239	Hospital	1(0.4%)	1(4.3%)	0(0)	30.00	<0.001
Ahn et al	74/1368	Hospital	14(1.0%)	3(4.1%)	11(0.8%)	5.04	0.03
Konda et al	107/790	30-day	12(1.3%)	3(2.8%)	9(1.1%)	2.46	0.107
Cardinale et al	222/1957	Hospital	3(0.1%)	1(0.5%)	2(0.1%)	4.41	0.28
Naruka et al	86/482	Hospital	11(1.9%)	2(2.3%)	9(1.9%)	1.25	0.226
Matesanz et al	12/162	Hospital	3(1.7%)	2(16.7%)	1(0.6%)	27.00	<0.001
Murphy et al	208/927	Hospital	24(2.1%)	7(3.3%)	17(1.8%)	1.84	0.181

Abbreviations: AKI, Acute kidney injury; RR, Relative risk.
